# A novel missense *PTEN* mutation identified in a patient with macrocephaly and developmental delay

**DOI:** 10.1038/s41439-019-0056-8

**Published:** 2019-05-23

**Authors:** Yuichi Ueno, Takashi Enokizono, Hiroko Fukushima, Tatsuyuki Ohto, Kazuo Imagawa, Mai Tanaka, Aiko Sakai, Hisato Suzuki, Tomoko Uehara, Toshiki Takenouchi, Kenjiro Kosaki, Hidetoshi Takada

**Affiliations:** 1 Department of Pediatrics, University of Tsukuba Hospital, Tsukuba, Japan; 2 Department of Child Health, Faculty of Medicine, University of Tsukuba, Tsukuba, Japan; 3 Center for Medical Genetics, Keio University School of Medicine, Tokyo, Japan

**Keywords:** Autism spectrum disorders, Genetics of the nervous system

## Abstract

Phosphatase and tensin homolog (PTEN) plays an important role in tumor suppression. A germline mutation in the *PTEN* gene induces not only PTEN hamartoma tumor syndrome, including Cowden syndrome, but also macrocephaly/autism syndrome. Here, we describe a boy with macrocephaly/autism syndrome harboring a novel missense heterozygous *PTEN* mutation, c.959T>C (p.Leu320Ser). Interestingly, a previously reported nonsense mutation resulting in p.Leu320X was found in Cowden syndrome patients. Our case may be suggestive of a genotype-phenotype correlation.

The phosphatase and tensin homolog (*PTEN*) gene (OMIM 601728), located on chromosome 10q23, is a tumor suppressor gene that has a significant role in cellular proliferation, migration, and apoptosis^1^. PTEN hamartoma tumor syndrome (PHTS), which comprises Cowden syndrome (CS; OMIM 158350), Bannayan–Riley–Ruvalcaba syndrome (BRRS; OMIM 153480), and Proteus-like syndrome, represents a spectrum of hamartomatous overgrowth manifestations associated with germline mutations in the *PTEN* gene^2^. CS is characterized by macrocephaly, trichilemmomas, facial acral keratosis, hamartoma, and an increased risk of certain cancers, particularly of the breast, uterus, and thyroid^3^. Under revised diagnostic criteria, the majority of CS diagnoses are made in adulthood because cutaneous findings or malignancies are rarely present before 20 years of age^4^. BRRS is typically diagnosed in childhood and is characterized by macrocephaly, hamartomas (including lipomas or intestinal polyps), penile freckling in males and developmental delays, including an increased risk of autism spectrum disorder (ASD). Because the clinical presentation of CS and BRRS is quite similar, they were considered to be a single disorder with variable phenotypic expression and age-related penetrance^5^. However, the clinical features of patients with *PTEN* mutations were quite variable, and presentations differed significantly even within the same family^6^.

*PTEN* mutations have been identified in children with macrocephaly associated with ASD and/or developmental delay without hamartomas known as macrocephaly/autism syndrome (OMIM 605309)^7,8^. However, the clinical features of these children have not been described in detail. We report here a 4-year-old boy with macrocephaly and developmental delay that had a novel missense mutation, NM_000314.4:c.959T>C [p.(Leu320Ser)], in the *PTEN* gene.

The patient was the first child of healthy nonconsanguineous Japanese parents. Family history was unremarkable. He was born after 38 weeks of gestation using vacuum extraction with a birth weight of 3.696 g (+1.7 SD), length of 52 cm (+1.4 SD) and head circumference of 37 cm (+2.6 SD). He could control his head at 5 months but was referred to our hospital for developmental delay and muscular hypotonia at 10 months of age.

His head circumference was 50.0 cm (+2.8 SD) at 10 months old. His anterior fontanelle was widely open with a prominent forehead, but he did not have any symptoms related to increased intracranial pressure, such as vomiting, seizures, or poor activity. He showed loose shoulder and heel to ear sign, suggesting general muscular hypotonus. Physical examination did not reveal heart murmur, organomegaly, cutaneous pigmentary lesions, or freckles on the penis. The overall developmental quotient was 60 by the Kinder Infant Development Scale^9^. Head MRI at 10 months showed white matter hyperintense areas on T2 weighted and fluid-attenuated inversion recovery (FLAIR) images, enlarged perivascular spaces, mild ventricular enlargement, and narrow posterior fossa (Fig. 1). The possibility of hydrocephalus was excluded after cisternography at 1 year and 1 month of age. He could walk alone at 1 year and 8 months of age. He spoke the first meaningful word at 1 year and 5 months and two phrases at 2 years and 6 months. The physical examination at 4 years and 2 months of age showed a weight of 17.4 kg (+0.7 SD), height of 104.0 cm (+0.7 SD), and head circumference of 56.5 cm (+3.6 SD). Chromosomal analysis showed 46,XY.

**Fig. 1:**
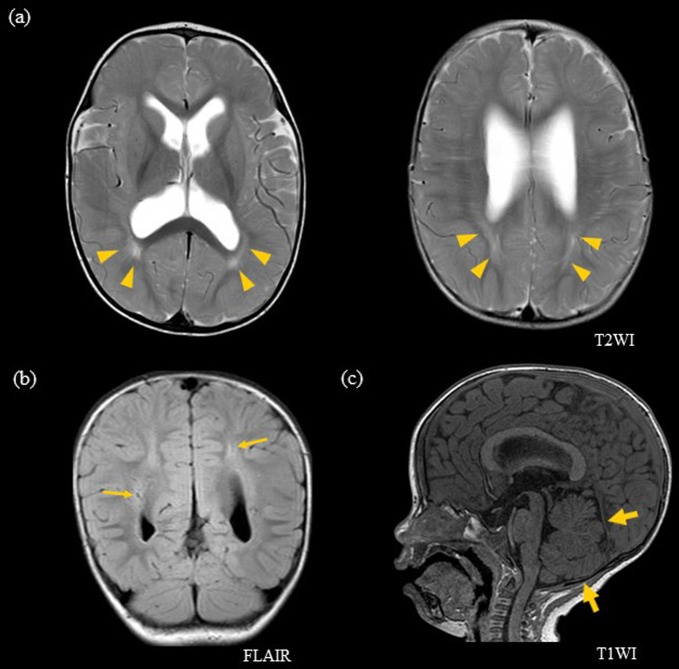
Head MRI at 10 months of age. **a** Axial T2-weighted image and **b** coronal FLAIR image show hyperintensity in the bilateral periventricular white matter regions (yellow arrowhead) and enlarged periventricular spaces (thin yellow arrow). **c** Sagittal T1-weighted image shows a narrow posterior fossa (broad yellow arrow)

A trio exome analysis was performed as described previously^10^. The research protocol was approved by the local Institutional Ethical Review Board of the University of Tsukuba and Keio University School of Medicine. Informed consent was obtained from the parents on the basis of the Declaration of Helsinki. Genomic DNA was extracted from peripheral blood leukocytes of the patient and his parents. Whole-exome sequencing using the SureSelectXT2 Human All Exon Kit V4 (Agilent Technologies, Santa Clara, CA) was performed on the HiSeq 1000 platform (Illumina, San Diego, CA) and showed a heterozygous missense mutation, NM_000314.4:c.959T>C, [p.(Leu320Ser)] in exon 8 of *PTEN* (OMIM #601728) that was confirmed by Sanger sequencing (Fig. 2). This mutation was found only in the patient and not in his parents, indicating that it was a de novo mutation. In silico analysis demonstrated that this mutation was likely pathogenic (SIFT: deleterious, score 0; PolyPhen: probably damaging, score 0.99; PROVEAN: damaging, score −4.2). To the best of our knowledge, this mutation has never been reported previously. Based on a literature review, the variants c.959T>A and c.959T>G, which result in p.Leu320X, have been previously reported in CS patients^4,11^.

**Fig. 2:**
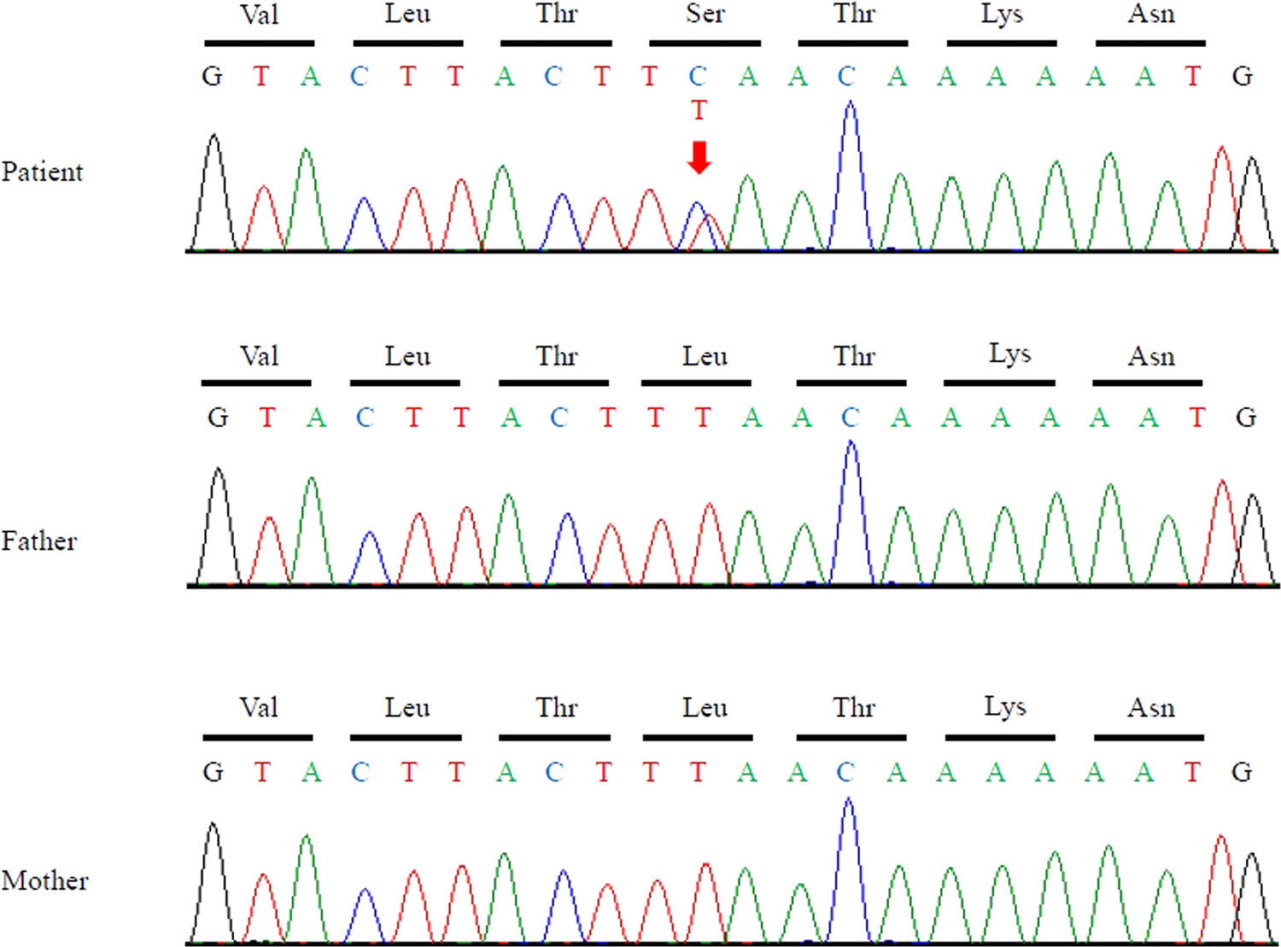
*PTEN* sequences of the patient and his parents. The patient has a heterozygous missense mutation, NM_000314.4: c.959T>C, [p.(Leu320Ser)] (arrow) in *PTEN* exon 8. This mutation was not found in his parents

In 2005, Butler et al.^8^ reported the presence of heterozygous germline mutations in the *PTEN* gene in three children with ASD and macrocephaly. Since then, germline *PTEN* pathogenic variants have been reported in 10–20% of ASD cases with macrocephaly. Furthermore, developmental delay, intellectual disability, and/or ASD have been recorded in 66% of children with pathogenic mutations in *PTEN*^3^.

Our patient presented with macrocephaly from birth, whereas developmental delay and muscular hypotonia without dermatological findings were noted from the infantile period. His brain MRI was consistent with previous reports of individuals with *PTEN* mutations who also presented with macrocephaly, developmental delay, and/or ASD^4^. Whole-exome sequencing identified a novel missense *PTEN* gene mutation, NM_000314.4:c.959T>C, [p.(Leu320Ser)], in the patient. However, he did not meet clinical criteria for BRRS or CS diagnoses. Although we diagnosed him with macrocephaly/autism syndrome provisionally, it is certainly possible that the patient will meet the criteria for CS/BRRS in the future.

Many of the mutations associated with CS and BRRS occur in exons 5, 7, and 8 of the *PTEN* gene^11^. Previous studies of CS/BRRS cohorts have failed to establish clear genotype-phenotype correlations^3^. Furthermore, Frazier et al.^12^ reported that germline heterozygous missense *PTEN* mutations were enriched in ASD and macrocephaly cases compared to their frequency in PHTS patients without ASD. Interestingly, the missense mutation Leu320Ser found in our patient was associated with macrocephaly and developmental delay, whereas the previously reported nonsense mutation Leu320X was found in a CS patient. Our case may be suggestive of a genotype-phenotype correlation even though we could not confirm this on the basis of a single case report. Further research is needed to ascertain this correlation.

The major concern in patients with *PTEN* mutations is a risk of malignancy. To date, however, a consensus cancer surveillance protocol has not been formally instituted, so all *PTEN* mutation carriers should adopt the cancer surveillance strategies proposed for patients with CS^2^. We started annual general surveillance and annual specific surveillance of thyroid cancer for future cancer risk in this patient.

In conclusion, we identified a novel missense *PTEN* gene mutation in a Japanese boy who presented with developmental delay and macrocephaly. We will continue to follow up with this patient regularly for the early detection of tumor development as well as to expand our knowledge of genotype-phenotype correlations in patients with pathological *PTEN* variants.

## Data Availability

The relevant data from this Data Report are hosted at the Human Genome Variation Database at 10.6084/m9.figshare.hgv.2579.
